# *IL-6/IL-10* mRNA expression ratio in tumor tissues predicts prognosis in gastric cancer patients without distant metastasis

**DOI:** 10.1038/s41598-022-24189-3

**Published:** 2022-11-12

**Authors:** Li Zhou, Chuangang Tang, Xiaoxin Li, Fang Feng

**Affiliations:** 1 Department of Oncology, Suzhou Ninth People′s Hospital, Suzhou Ninth Hospital Affiliated to Soochow University, Suzhou, 215200 China; 2 Department of General Surgery, Xuzhou Central Hospital, The Affiliated Xuzhou Hospital of Medical College of Southeast University, Xuzhou, 221009 China; 3 Department of Breast Surgery, Xuzhou Central Hospital, The Affiliated Xuzhou Hospital of Medical College of Southeast University, Xuzhou, 221009 China; 4 Department of Pathology, Xuzhou Central Hospital, The Affiliated Xuzhou Hospital of Medical College of Southeast University, Xuzhou, 221009 China

**Keywords:** Gastrointestinal cancer, Prognostic markers

## Abstract

There was growing evidence that inflammatory responses played significant roles in malignancies. However, the impact of pro-inflammatory-to-anti-inflammatory factor ratio in tumor tissues has not been investigated in gastric cancer (GC) yet. We collected patient data from The Cancer Genome Atlas (TCGA) database. A total of 270 stomach adenocarcinoma (STAD) patients without distant metastasis were included in the study. After screening 12 candidate pro-inflammatory-to-anti-inflammatory pairs, only the *IL-6/IL-10* mRNA expression ratio in tumor tissues had a significant effect on overall survival (OS) of STAD patients (P = 0.014). X-tile analysis showed that the greatest survival differences were obtained when the cutoff value of *IL-6/IL-10* mRNA expression ratio was set at 1.3 and 5.5. With the low-ratio group (*IL-6/IL-10* mRNA expression ratio: < 1.3) as reference, OS time for both the medium-ratio group (*IL-6/IL-10* mRNA expression ratio: 1.3–5.5) and the high-ratio group (*IL-6/IL-10* mRNA expression ratio: > 5.5) was significantly shorter (P < 0.05). Multivariate Cox regression analyses indicated that *IL-6/IL-10* mRNA expression ratio was an independent prognostic factor for OS and disease-specific survival (DSS). These findings provided a novel and powerful tool for a more rational management of GC patients.

## Introduction

Gastric cancer (GC) is one of the most common malignancies of the digestive system^1,2^. Multiple etiological factors, including Helicobacter pylori (HP) infection, dietary habits, and genetics, are considered to be closely associated with the tumorigenesis and development of GC^3,4^. Based on the immunoblot-based data, HP infection accounted for 89% of the global GC^5^. It was estimated that there were approximately 1.2 million new GC cases worldwide in 2017^6^, indicating that one million cases may be related to HP infection. Surgical resection alone or combined with radiotherapy and chemotherapy is a mainstay of treatment and the only potential cure for GC. However, the overall prognosis of GC is still not optimistic. Due to the difficulty of early detection and diagnosis, GC is usually diagnosed in intermediate and late stages with a 5-year survival rate of < 20%^7^. Therefore, there is an urgent need for further understanding the molecular mechanisms underlying the tumor occurrence and development and exploring novel biomarkers.

In normal physiological conditions, inflammatory response system keeps various chemokines and cytokines such as pro-inflammatory factors and anti-inflammatory factors in balance. Previous studies of inflammatory responses primarily focused in the field of injury, infection, autoimmune diseases, hematological disorders and malignancies^8–11^. There was growing evidence that inflammatory responses also played significant roles in solid malignancies. For example, interleukin-6 (IL-6), a common pro-inflammatory factor, promoted tumor cell proliferation, invasiveness, and metastasis, and affected the clinical prognosis in various solid malignancies including gastric cancer^12–15^. However, pro-inflammatory factors did not always play the roles of “pro-oncogenic factors”. In early-stage invasive breast cancer patients, high expression of IL-6 was closely associated with favorable clinical outcomes^16^. Similarly, IL-10, a common anti-inflammatory factor, played a dual role as a pro-oncogenic factor as well as a tumor suppressor in cervical cancer^17^. Thus, the balance disorder of pro-inflammatory factors and anti-inflammatory factors may break the tumor inflammatory status and consequently participate in the tumor occurrence and development. Liu et al.^18^ found that serum *IL-6/IL-1Ra* ratio could predict the response of patients with metastatic non-small cell lung cancer to chemotherapy. Terracciano et al.^19^ showed that soluble IL-6 receptor to IL-6 (*sIL-6R/IL-6*) ratio in serum was an effective tumor marker for prostate cancer. A recent randomized clinical trial demonstrated that perioperative inflammatory factors were critical prognostic indicators for GC, and anti-inflammatory therapy might increase the potential clinical benefits^20^. However, the impact of pro-inflammatory-to-anti-inflammatory factor ratio in tumor tissues has not been investigated in solid malignancies including GC yet.

In the present study, we used data from The Cancer Genome Atlas (TCGA) database to investigate whether pro-inflammatory-to-anti-inflammatory factor ratio in tumor tissues has an effect on the prognosis of GC patients.

## Results

**Figure 1:**
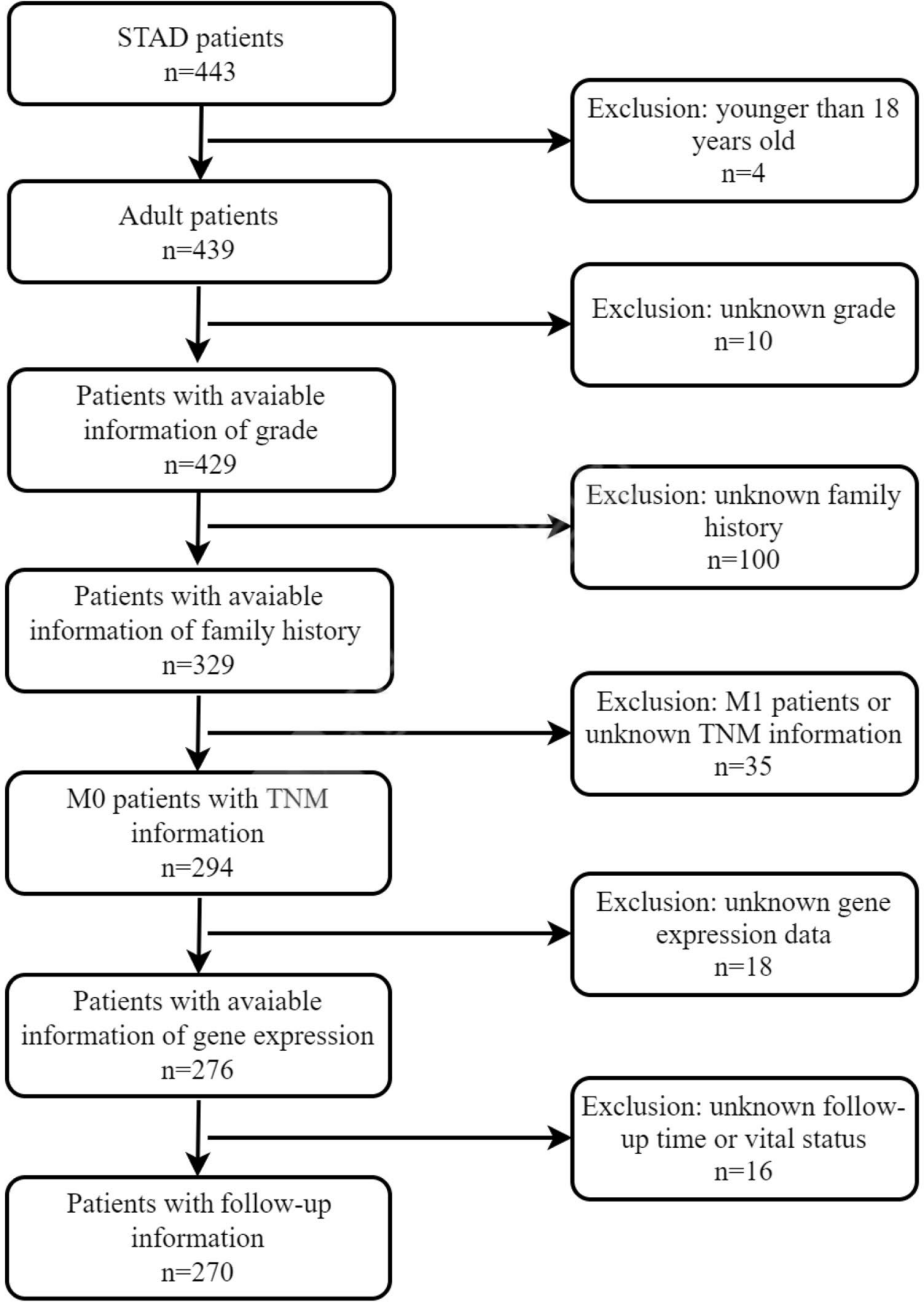
Screening flowchart.

After rigorous screening progress, a total of 270 stomach adenocarcinoma (STAD) patients without distant metastasis were included in the study (Fig. 1). Age ranged from 30 to 90 years old with the median age of 67 (Table 1). There were 171 male (63.3%) and 99 female (36.7%) cases. The great majority (94.1%) did not have a family history of gastric cancer. Entire cohort showed a predominance of tumors with grade II (90, 33.3%) and III (174, 64.5%) while only 6 cases (2.2%) had tumors with grade I. According to the AJCC staging system, 127 cases were classified as stage I or II, and 143 were classified as stage III or IV. The follow-up period ranged from 0 to 3720 days with the median time of 500 days. During the follow-up period, there were 105 deaths.

**Table 1:** Baseline Information of STAD Patients without Distant Metastasis.

Variables	Number (%)
Total	270 (100%)
**Gender**	
Male	171 (63.3%)
Female	99 (36.7%)
**Age, years**	
< 65	120 (44.4%)
≥ 65	150 (55.6%)
Median (range)	67 (30–90)
**Family history**	
No	254 (94.1%)
Yes	16 (5.9%)
**Grade**	
I	6 (2.2%)
II	90 (33.3%)
III	174 (64.5%)
AJCC stage*	
I + II	127 (47.1%)
III + IV	143 (52.9%)
**Vital status**	
Dead	105 (38.9%)
Live	165 (61.1%)

*According to the sixth edition of AJCC staging system for gastric cancer.

**Figure 2:**
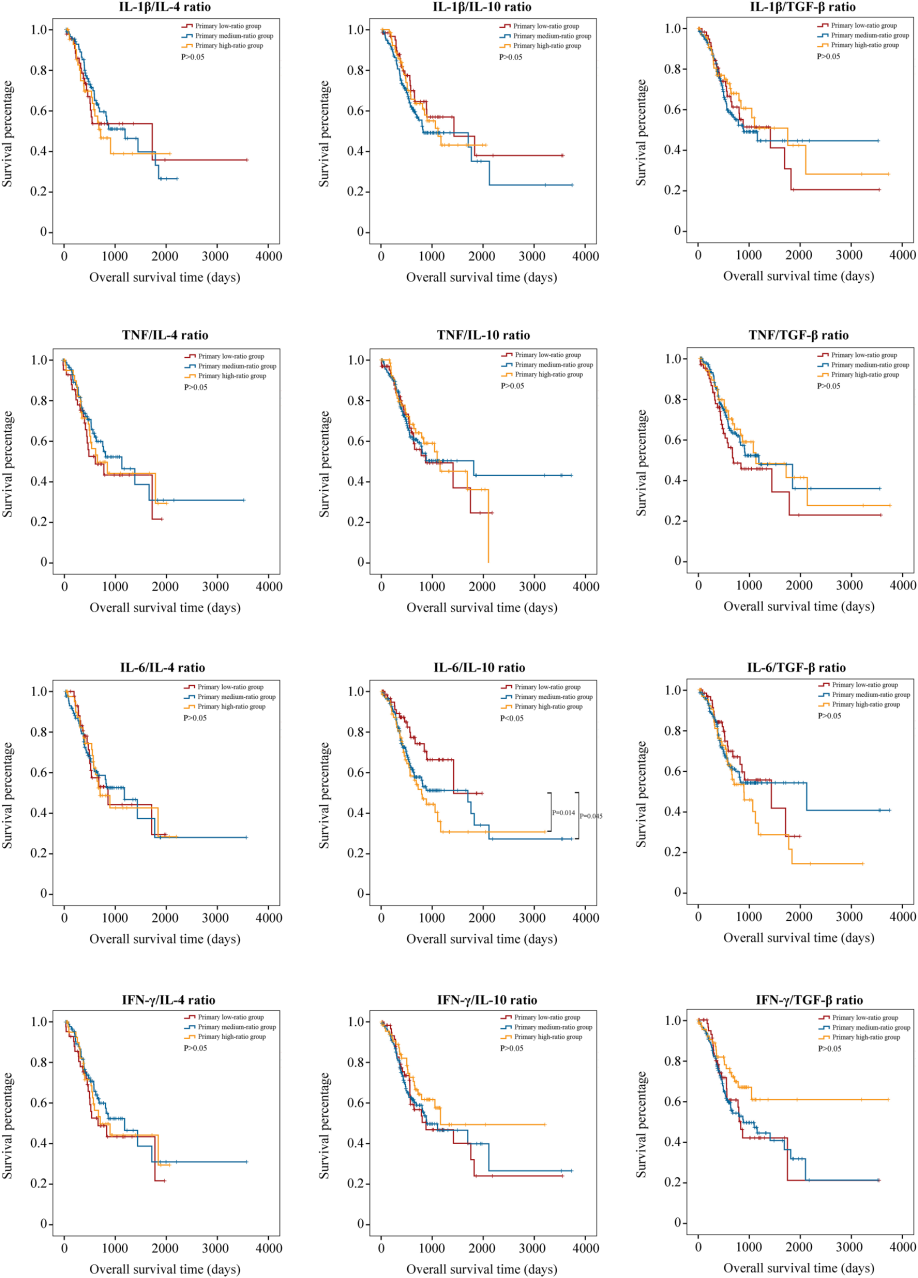
The prognostic values of common pro-inflammatory-to-anti-inflammatory factor mRNA expression ratio.

Kaplan–Meier survival analysis of common pro-inflammatory-to-anti-inflammatory factor ratio was performed, including *IL-1β/IL-4* mRNA expression ratio, *IL-1β/IL-10* mRNA expression ratio, *IL-1β/TGF-β* mRNA expression ratio, *TNF/IL-4* mRNA expression ratio, *TNF/IL-10* mRNA expression ratio, *TNF/TGF-β* mRNA expression ratio, *IL-6/IL-4* mRNA expression ratio, *IL-6/IL-10* mRNA expression ratio, *IL-6/TGF-β* mRNA expression ratio, *IFN-γ/IL-4* mRNA expression ratio, *IFN-γ/IL-10* mRNA expression ratio, and *IFN-γ/TGF-β* mRNA expression ratio. Entire cohort was ordered from a small ratio to a large ratio. The top quartile was named primary low-ratio group; the bottom quartile was named primary high-ratio group; the remaining patients were named primary medium-ratio group. Only the *IL-6/IL-10* mRNA expression ratio had a significant effect on OS of STAD (Fig. 2). The prognosis of the primary high-ratio group was much worse than that of the primary low-ratio group (P = 0.014).

**Figure 3:**
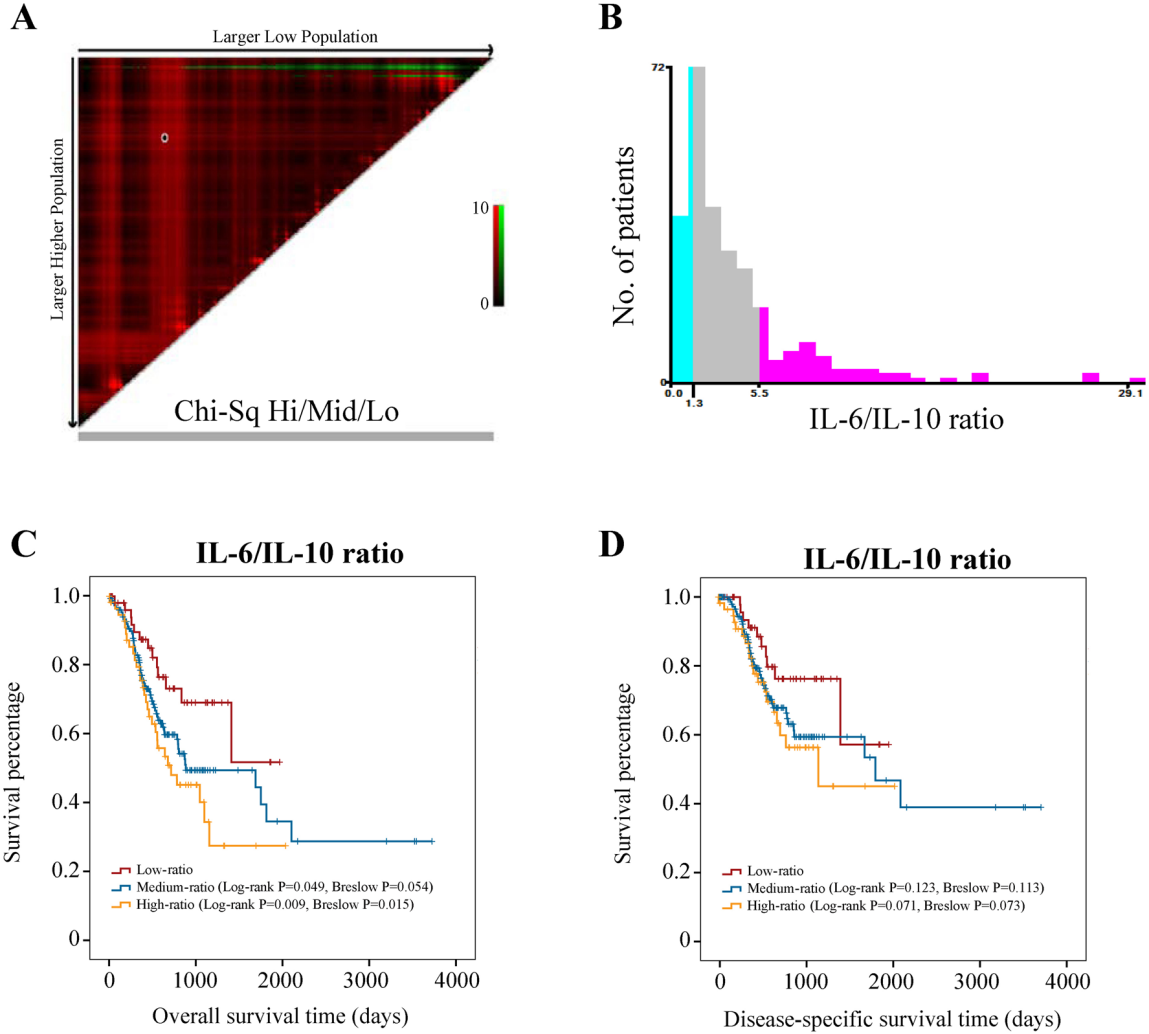
The optimal cutoff value of *IL-6/IL-10* mRNA expression ratio determined by X-tile. (**A-B**) The greatest survival differences were obtained when the cutoff value of *IL-6/IL-10* mRNA expression ratio was set at 1.3 and 5.5. (**C**) Kaplan–Meier overall survival curves for the low-, medium-, and high-ratio group. (**D**) Kaplan–Meier disease-specific survival curves for the low-, medium-, and high-ratio group.

X-tile analysis showed that the greatest survival differences were obtained when the cutoff value of *IL-6/IL-10* mRNA expression ratio was set at 1.3 and 5.5 (Fig. 3A,B). Thus, all the cases were renamed as follows: low-ratio group (ratio value: 0–1.3), medium-ratio group (ratio value: 1.3–5.5), and high-ratio group (ratio value > 5.5). With the low-ratio group as reference, OS time for the medium-ratio group [HR = 1.826, 95% CI = (1.004, 3.323), Log-rank P = 0.049] and the high-ratio group [HR = 2.387, 95% CI = (1.240, 4.596), Log-rank P = 0.009] was significantly shorter (Fig. 3C). 5-year survival rates of the low-, medium-, and high-ratio group were 51.9%, 34.7%, and 27.6%, respectively. No significant impact of *IL-6/IL-10* mRNA expression ratio on DSS was observed (Fig. 3D, P > 0.05). However, the survival difference between the low-ratio group and the high-ratio group nearly reached statistical significance (Log-rank P = 0.071).

The relationship between the *IL-6/IL-10* mRNA expression ratio and baseline variables was further investigated. As shown in Table 2, the low-ratio group had higher proportions of tumors with grade III while the high-ratio group had higher proportions of tumors with grade II (P = 0.002). Regarding gender, age, family history, and AJCC stage distribution, no statistical significance existed. We next performed univariate and multivariate Cox regression analyses to identify independent prognostic factors for OS and DSS (Tables 3 and 4). *IL-6/IL-10* mRNA expression ratio (low-ratio group vs. high-ratio group) and AJCC stage (I + II vs. III + IV) were independent prognostic factors for OS. Gender, *IL-6/IL-10* mRNA expression ratio (low-ratio group vs. high-ratio group), and AJCC stage (I + II vs. III + IV) were independent prognostic factors for DSS.

**Table 2:** Comparison between Low and High *IL-6/IL-10* mRNA expression ratio Group.

Variables	Low-ratio group (n = 57)	High-ratio group (n = 58)	P
**Gender**			0.756
Male	36	35	
Female	21	23	
**Age**			0.077
< 65	32	23	
≥ 65	25	35	
**Family history**			0.714
No	54	54	
Yes	3	4	
Grade*			0.002
I	1	2	
II	16	33	
III	40	23	
**AJCC stage**			0.647
I + II	26	24	
III + IV	31	34	

*Fisher exact test.

**Table 3:** Univariate Analysis for OS and DSS of STAD Patients without Distant Metastasis.

Variables	OS		DSS	
	HR (95% CI)	Log-rank P	HR (95% CI)	Log-rank P
**Gender**				
Male	Reference		Reference	
Female	0.649 (0.425–0.993)	0.046	0.434 (0.250–0.756)	0.003
**Age**				
< 65	Reference		Reference	
≥ 65	1.509 (1.013–2.249)	0.043	1.216 (0.767–1.927)	0.405
**Family history**				
No	Reference		Reference	
Yes	1.006 (0.488–2.071)	0.987	0.869 (0.350–2.157)	0.763
***IL-6/IL-10* mRNA expression ratio**				
Low	Reference		Reference	
Medium	1.826 (1.004–3.323)	0.049	1.714 (0.864–3.403)	0.123
High	2.387 (1.240–4.596)	0.009	2.036 (0.942–4.362)	0.071
**Grade**				
I	Reference		Reference	
II	1.126 (0.270–4.705)	0.871	0.835 (0.197–3.537)	0.806
III	1.495 (0.366–6.102)	0.576	1.012 (0.246–4.167)	0.987
**AJCC stage**				
I + II	Reference		Reference	
III + IV	1.855 (1.236–2.738)	0.003	1.967 (1.211–3.197)	0.006

**Table 4:** Multivariate Analysis for OS and DSS of STAD Patients without Distant Metastasis.

Variables	OS		DSS	
	HR (95% CI)	Log-rank P	HR (95% CI)	Log-rank P
**Gender**				
Male	Reference		Reference	
Female	0.662 (0.430–1.017)	0.060	0.449 (0.257–0.785)	0.005
**Age**				
< 65	Reference		Reference	
≥ 65	1.464 (0.972–2.204)	0.068	1.173 (0.729–1.887)	0.511
**Family history**				
No	Reference		Reference	
Yes	0.869 (0.415–1.818)	0.710	0.721 (0.281–1.847)	0.495
***IL-6/IL-10* mRNA expression ratio**				
Low	Reference		Reference	
Medium	1.689 (0.923–3.091)	0.089	1.659 (0.827–3.329)	0.154
High	2.461 (1.255–4.825)	0.009	2.203 (1.000–4.854)	0.050
**Grade**				
I	Reference		Reference	
II	1.071 (0.251–4.571)	0.926	0.703 (0.159–3.095)	0.641
III	1.669 (0.398–7.002)	0.484	0.950 (0.221–4.083)	0.945
**AJCC stage**				
I + II	Reference		Reference	
III + IV	1.696 (1.121–2.565)	0.012	1.776 (1.083–2.913)	0.023

## Methods

### Patient data

Data for the mRNA expression levels of inflammatory factors (level 3) in tumor tissues and baseline clinical characteristics were obtained from TCGA database (https://tcga-data.nci.nih.gov/tcga/), which was the largest cancer gene and clinical information database. Common pro-inflammatory factors included IL-1β, tumor necrosis factor (TNF), IL-6, and interferon-gamma (IFN-γ). Common anti-inflammatory factors included IL-4, IL-10, and transforming growth factor-beta (TGF-β). Baseline clinical characteristics included gender, age, family history, grade, American Joint Committee on Cancer (AJCC) stage, follow-up period, and vital status. Inclusion criteria were as follows: (1) 18 years old or elder; (2) definite information of grade and family history; (3) definite tumor-node-metastasis (TNM) information according to the sixth edition of AJCC staging system for GC; (4) complete data of mRNA expression; (5) available follow-up data. Exclusion criteria were as follows: (1) distant metastasis; (2) Grade information was labeled as Gx; (3) labeled as unknown or blank; (4) Unknown cause of death. The primary endpoints of the study included overall survival (OS) and disease-specific survival (DSS). OS refers to the period between the date of initial diagnosis and the last follow-up date or the date of all-cause death. DSS refers to the period between the date of initial diagnosis and the last follow-up date or the date of gastric cancer-related death. The study protocol was approved by the Ethics Committee of Suzhou Ninth People’s Hospital.

### Survival analysis

A chi-square test was used for comparison between groups. Fisher exact test was used when the number of samples was small. Kaplan–Meier analysis, Log-rank test, and Breslow test were used for comparison of survival differences. Univariate and multivariate Cox regression analyses were used for assessing the effect of various factors on the prognosis. The above statistical methods were completed using SPSS 22.0 (SPSS Inc. Chicago, IL).

X-tile software (version 3.6.1, Yale University School of Medicine), an algorithm that could try every cutoff value and filter out the results automatically, was used to explore the optimal cutoff value of pro-inflammatory-to-anti-inflammatory factor ratio. P-values less than 0.05 were considered statistically significant.

## Discussion

The association between inflammation and cancer was first noted in thenineteenth century^21^. Substantial evidence has revealed that inflammatory cells and factors were important components of the tumor microenvironment, which activated or inhibited various signaling pathways to influence tumor cell behavior. In the present study, our results demonstrated for the first time that the *IL-6/IL-10* mRNA expression ratio was an independent prognostic factor for GC patients without distant metastasis. The optimal cutoff value of *IL-6/IL-10* mRNA expression ratio was 1.3 and 5.5. Patients with *IL-6/IL-10* mRNA expression ratio > 5.5 had the highest risk of death. These findings identified novel biomarkers for GC and provided novel insights into molecular mechanisms underlying the tumorigenesis and development.

As shown in Fig. 3C, the survival gap between the low-ratio group and the medium-ratio group was obviously larger than that between the medium-ratio group and the high-ratio group. Although the survival time in the high-ratio group was shorter, there was no statistical difference between the medium-ratio group and the high-ratio group (P = 0.241). These may suggest that the association between the *IL-6/IL-10* mRNA expression ratio and the degree of tumor malignancy is not a strong linear positive correlation. Therefore, we speculate that the risk of death increases with the greater values of *IL-6/IL-10* mRNA expression ratio at first. When the *IL-6/IL-10* mRNA expression ratio arrives at a certain value, IL-6 and IL-10 show the greatest synergistic effects in promoting GC development. However, after the certain value, the trend towards increased risk of death gradually slows or even decreases. This also explained why the medium-ratio group had a higher proportion of tumors with grade III.

IL-6, first discovered in 1986, is a well-known inflammatory cytokine with pleiotropic characteristics^22–24^. Upon binding of IL-6R, IL-6 exerts its function mainly through MAPK signaling pathway and JAK-STAT3 signaling pathway^25^. Liu et al. showed that the increased levels of IL-6 in tumor tissues were associated with worse clinical outcomes in young and elderly patients with GC, which was suggestive of the prognostic value of IL-6 in tumor tissues.

IL-10, mainly secreted by stimulated myeloid cells and lymphocytes, usually inhibits NFκB-mediated signal transduction and ultimately terminates the inflammatory response^26,27^. One clinical trial revealed that serum IL-10 was an independent prognostic factor in patients with GC^28^. However, no clinical association between the expression levels of IL-10 in tumor tissues and the prognosis of GC has been reported yet.

*IL-6/IL-10* ratio was a common indicator in both neoplastic and nonneoplastic diseases. *IL-6/IL-10* ratio in the aqueous humor and the vitreous could be used as an early diagnostic tool for vitreoretinal lymphoma (VRL)^29^. In primary open-angle glaucoma (POAG), serum *IL-6/IL-10* ratio contributed to discriminate the disease progression^30^. Serum *IL-6/IL-10* ratio was also used to evaluate the severity of pneumocystis pneumonia in HIV/AIDS patients^31^. In the present study, we expanded the application field of *IL-6/IL-10* mRNA expression ratio into tumor tissues. To obtain potential and useful prognostic value as accurately as possible, four common pro-inflammatory factors and three anti-inflammatory factors were included for the analysis, and all the enrolled individuals were divided into three groups. However, the results demonstrated that only *IL-6/IL-10* mRNA expression ratio had a prognostic value while the other 11 indicators did not. This may be attributed to the relatively small sample size. Compared to low and medium *IFN-γ/TGF-β* mRNA expression ratio group, high *IFN-γ/TGF-β* mRNA expression ratio group had a trend to obtain longer periods of OS although the survival difference did not reach statistical significance (Fig. 2).

There were several limitations in the study. First, all the data from TCGA were retrospective, potentially resulting in selection and follow-up bias. Second, the strict inclusion and exclusion criteria caused a reduction in the sample size to some extent, which may represent a highly selective study population. Third, there was no external data to verify our findings. Further multicenter, prospective clinical trials are still required. Fourth, lack of information concerning recurrence and postoperative complications in the TCGA database restricts further analysis and limits the possibilities of the application of *IL-6/IL-10* mRNA expression ratio.

In sum, *IL-6/IL-10* mRNA expression ratio was identified as an independent prognostic factor for GC patients without distant metastasis for the first time, which provided a novel and powerful tool for a more rational management of GC patients.

## Data Availability

The datasets generated and analyzed during the current study are available in the TCGA database (https://portal.gdc.cancer.gov/).
